# Cooperative vehicles for robust traffic congestion reduction: An analysis based on algorithmic, environmental and agent behavioral factors

**DOI:** 10.1371/journal.pone.0182621

**Published:** 2017-08-08

**Authors:** Prajakta Desai, Seng W. Loke, Aniruddha Desai

**Affiliations:** 1 Department of Computer Science and Information Technology, La Trobe University, Victoria, Australia; 2 School of Information Technology, Deakin University, Victoria, Australia; Beihang University, CHINA

## Abstract

Traffic congestion continues to be a persistent problem throughout the world. As vehicle-to-vehicle communication develops, there is an opportunity of using cooperation among close proximity vehicles to tackle the congestion problem. The intuition is that if vehicles could cooperate opportunistically when they come close enough to each other, they could, in effect, spread themselves out among alternative routes so that vehicles do not all jam up on the same roads. Our previous work proposed a decentralized multiagent based vehicular congestion management algorithm entitled Congestion Avoidance and Route Allocation using Virtual Agent Negotiation (CARAVAN), wherein the vehicles acting as intelligent agents perform cooperative route allocation using inter-vehicular communication. This paper focuses on evaluating the practical applicability of this approach by testing its robustness and performance (in terms of travel time reduction), across variations in: (a) *environmental parameters* such as road network topology and configuration; (b) *algorithmic parameters* such as vehicle agent preferences and route cost/preference multipliers; and (c) *agent-related parameters* such as equipped/non-equipped vehicles and compliant/non-compliant agents. Overall, the results demonstrate the adaptability and robustness of the decentralized cooperative vehicles approach to providing global travel time reduction using simple local coordination strategies.

## Introduction

Our proposed co-operative route allocation approach entitled, Congestion Avoidance and Route Allocation using Virtual Agent Negotiation (CARAVAN), first introduced in [1], employs inter-agent coordination and negotiation (via vehicular ad hoc networks or VANETs) as the basis of cooperation in which the routes are traded by the vehicle agents to arrive at an efficient route allocation. Also, other work such as [2] demonstrated the use of inter-vehicle communication for detecting as well as controlling congestion. In our previous work in [1], we showed that with CARAVAN, effective global traffic management can emerge from local negotiations among agents (having specific route preferences) at disparate road junctions. However, our previous work did not answer important questions regarding the robustness of the approach, as algorithmic, environmental and agent-specific parameters are varied: *how would the number and placement of junctions and the road segment capacity affect emergent traffic flows? What if drivers do not follow the system’s recommendations and only some percentage of vehicles are equipped with CARAVAN? What traffic behaviors would emerge if the system favors drivers with only specific route preferences?* In this paper, we address these questions by comprehensively analyzing the effects of the aforementioned parameters on the performance of CARAVAN in terms of travel time reduction. This work provides insights into the emergent behaviors of the cooperative self-organizing multiagent vehicle models under different conditions enabled by the virtual negotiation deals. The two main contributions of this paper are: (i) demonstrating the *robustness* (over an extensive range of conditions) of the local coordination mechanism used in the approach to regulate the overall traffic; and (ii) demonstrating the effect of factors such as driver preferences and response, road topology and configuration on the route allocation in terms of travel time reduction.

The rest of the paper is organized as follows. We first review work on multiagent based cooperative traffic congestion management. Then, we provide an overview of CARAVAN, and describe the virtual negotiation mechanism design and the algorithmic parameters used in CARAVAN. A comprehensive evaluation of CARAVAN os then presented, and then we conclude.

## Related work

### Multiagent based congestion management

We classify multiagent systems or MAS based congestion management approaches as:

1. *Cooperation and Self-organization based techniques*: MAS techniques can be used to detect and avoid traffic congestion. MAS based techniques such as ant pheromone, honey-bee foraging and fish schooling are inspired from the peculiar features of species (ants, honey-bees, fish). These techniques aid in traffic flow forecasting and traffic organization with indirect communication leading to well-coordinated movement of vehicles. The ant-pheromone technique proposed in[3], involves vehicles depositing digital pheromone (such as speed and acceleration) to build a dynamically weighted network graph depicting the areas and levels of congestion. The Adaptive and Cooperative Traffic light Agent Model (ACTAM) described in [4], has Intelligent Intersection Agents (IIA) capable of storing data and inter-communicating, learning from past/current traffic patterns and forecasting traffic states.
2. *Negotiation based Techniques*: The conflicting objectives of driver satisfaction and network stability can be handled using multiagent negotiation techniques which can lead to good network performance and increase in driver satisfaction by allocating drivers evenly along the network. For example, the Urban Traffic Control (UTC) technique in [5] consists of Roadside Agents (RSA), Intelligent Traffic Signaling Agent (ITSA) and Authority Agent (supervises and controls several ITSAs). Using the roadside information (collected by RSA) ITSA devises traffic control strategies, estimates the traffic state and is also capable of resolving conflicts via cooperation and negotiation. Another cooperative MAS based route guidance approach in [6] has three types of agents for: (i) providing traffic information; (ii) satisfying drivers' route choice; and (iii) focusing on overall network stability.
3. *Driver-behavior based model*: Drivers can be given intelligent advice to improve their ability to cope with congestion. The work in [7] simulates various scenarios with varying driver feedback/conformance, real-time information provisioning and driver’s ability to observe the local traffic conditions. The authors in [8] describe an Adaptive Route Advisor that generates routes for the drivers by adapting its recommendation via continuous driver interaction.

The system performance in the traffic management scenarios depends on collective action, i.e. the number of agents (the vehicles) taking a particular action (such as route selection) rather than the intrinsic value of a particular action [9]. Furthermore, the conflicting objectives of the agents can be resolved using negotiation strategies and the effectiveness of the route guidance system is enhanced when the agent preferences are taken into consideration. A detailed review of various congestion management techniques is in [10].

### Application of local coordination and self-organization based MAS techniques

In a limited resource environment the computation requirements of the coordination technique are important. Furthermore, the factors to be considered are number of agents, heterogeneity of agents, agent complexity, extent of agent interaction, degree of dynamism and distributivity. Three types of coordination mechanisms described in [11] are: (i) *conventions*–they are the simplest form of coordination, agents interact by means of “social rules” describing ways for agents to interact with other agents; (ii) *communication*–involves information-sharing bound by timeliness and bandwidth constraints; and (iii) *learning*–these evolve coordinated policies within uncertain state spaces.

The approaches below elaborate on how local coordination and self-organisation in the form of local knowledge, local interaction and local decision-making can contribute towards satisfying the global aims of the system. The approaches also describe how coordination can emerge from the autonomous and dynamic behaviour of the autonomous intelligent agents.

In [12] is proposed a hierarchical local coordination solution to the global stabilisation problem in context of the electricity domain. The agents interact and use local knowledge towards stabilising the global energy consumption of an electricity network. In this approach, the agents are arranged in a hierarchy and each of the child nodes generate unique energy consumption plans. An aggregate of these plans is sent to their respective parent nodes–this is done as a recursive process. All the plans thus obtained from the nodes are aggregated and passed down to each of the nodes in the hierarchy. In successive rounds, a plan which is least deviating from the previous aggregate plan is selected–this is called the stabilising goal. Thus, the agents at each level of the tree, select a plan that satisfies this goal locally, and also eliminates the less promising options. Experimental results with this hierarchical coordination suggested reduction in the deviation of the global plan in the range 36.54–78.71%compared to greedy agents.

In [13] is proposed the integration of the Ant Colony Intelligence (ACI) approach with MAS for dynamic manufacturing scheduling. The system contains entities such as job, machines and order with their respective goals. In the proposed approach, the machine agent acts as an ACI agent for solving the task sequencing problem. Every machine is regarded as an ant and the machine with the shortest processing time will have the highest pheromone value. After an operation is processed, the pheromone value of the machine agent is updated depending upon its current local status and the total processing time of the machine entity. Similarly, the job agent is also regarded as an ACI agent. The job agent with closest due date of its job has the highest initial pheromone level; and delayed jobs possess a higher pheromone level to attract the machine agents. Experimental results showed that the MAS with ACI outperformed the MAS with FIFO in regards to measurements such as reduced buffer size, reduced mean flow time, and reduced tardiness. The results demonstrated that local coordination results in efficient job sequencing and machine utilisation, and can adapt dynamically to the changing circumstances on the shop floor.

In [14] is described a cooperative satisficing MAS based approach to free flight for air traffic management. In this approach, which is based on the satisficing game theory during every round, an agent is assumed to have a current list of information about every plane within 50 miles. In this approach, the aircrafts adopt a satisficing approach which allows conditional altruism which means taking into consideration desires of others while making their own decisions. This is to ensure efficiency (arriving at the destination) and safety (avoiding collisions). The final solution selected is the option with maximum difference between the selectability and rejectability criteria. The selectability and rejectability criteria are designed to identify solutions which are good enough. The selectability criteria are the ones which allow the agents to achieve their goal. Aircraft headings and a particular option allowing the agent to follow its nominal route more closely are defined as the selectability criteria in this context. Any option which will lead to probable conflict with another plane is the rejectability criteria. The aircrafts rank each other using information on the current velocity, heading, altitude, desired direction, time in the air and current delay time.

It can be seen from the above examples, how distributed agents can cooperate locally either directly (with communication) or indirectly (without communication) and still regulate the overall results in the form of global optimization or in the form of a satisficing solution (for a resource, time and communication constrained environment).

## An overview of CARAVAN

CARAVAN is a traffic congestion management algorithm which uses inter-agent coordination and negotiation strategies for cooperative route allocation. If the vehicles are made aware of each other’s intentions (route choices), they can adjust their own routes and cooperate with each other to minimize any conflicting route choice decisions. The algorithm aims to achieve this by forming a number of localized interaction groups of vehicles which make cooperative route choices before they approach the road junctions resulting in coordinated movement of vehicles as they pass the designated junctions. These local interactions result in the self-organization of the vehicles along the road segments around the junctions, which in turn leads to an overall regulation of the traffic. The junctions act as the decision points to distribute the vehicles along the alternative routes leading from it. The local interaction between the vehicles is enabled by the inter-vehicular communication used to exchange their route preference information. The route allocation problem entails reducing the overall cost of vehicle traversal in terms of travel time and for the driver it entails traversing on his/her preferred route. These objectives which can be conflicting at times are formulated in an equation to evaluate the ‘utility’ of an allocation given as follows:

$$u_{i}(\sigma )=p*p_{i,r}(\sigma )-c*c_{i,r}(n_{r}(\sigma ))$$ (1)

where, *p* and *c* are the constant multipliers, *p*_*i*,*r*_(*σ*) corresponds to the preference utility index (PUI) of agent *i* in an allocation *σ* for route *r* and, *c*_*i*,*r*_(*n*_*r*_(*σ*)) corresponds to the cost of allocation *σ* experienced by agent *i* due to *n*_*r*_(*σ*) number of agents on route *r*.

The PUI of a route is the compact representation of the ranked agent preferences. It depends on the Preference Utility Weight (PUW) derived from various factors such as travel time and distance tolerance of an agent, deviation index (degree of flexibility to deviate from the preferred route), availability of the alternative route, compliance pattern of the driver and the level of familiarity with the route as explained in [1]. The cost of the allocation depends on the number of vehicles taking the route and the average travel time on that route.

The allocation is altered by every agent individually and iteratively per round of the virtual deals. CARAVAN addresses the problem of route allocation using Multiagent Based Route Allocation (MARA) technique [15] consisting of a finite number of agents (vehicles) and a finite number of indivisible but sharable resources (routes). As shown in (1), the utility function for CARAVAN is a multi-objective function with objectives to maximize the preference utility index factor, whose value is between 0 and 1, and to minimize the cost factor, whose value is a positive integer. A multi-objective problem is of the form given as:

$$\underset{x∊S}{\min}\left\{f_{1}(x),f_{2}(x)\ldots f_{k}(x)\right\}$$

where, *f*_*1*_*(x)…f*_*k*_*(x)* are the functions to be minimized and S is the solution space. Clearly, the values of the objective functions of the PUI and the cost do not fall within the same range. It is therefore inappropriate to compare and use these values directly in an equation. Hence, it is necessary to normalize these values before they are compared. Function transform methods are used for scalarization of the utility function. Scalarization of a multi-objective utility function means to transform it into a single-objective utility problem [16]. The functions for evaluating the preference utility index and the cost are each normalized using the transform function in (2) given in [17], and then used in the utility function in (1).

$$F^{Trans}=(F_{i(x)}-F_{i}^{0})/(F_{i}^{m}-F_{i}^{0})$$ (2)

where, *F*^*Trans*^ is the final normalized value, *F*_***i***(***x***)_ is the value of the function in the current iteration, $F_{i}^{m}$ is the maximum value and $F_{i}^{0}$ is the minimum value of the function evaluated till the current iteration. The above function yields a maximum value of 1 and minimum value of 0.

To solve the route allocation problem in CARAVAN, we use the weighted sum method, for evaluating the utility (in (1)), which is given as below:

$$\min_{x∊S}\sum_{i=1}^{k}w_{i}f_{i}(x)$$ (3)

where, the weights of the objectives, *w*_*i*_ > 0.

Here, the aim is to maximize the preference utility index function, say *f*_*1*_*(x)* and minimize the cost, say *f*_*2*_*(x)*; and hence as per the convention of (3) is represented as minimizing the negative of the function *f*_*1*_*(x)*.

In a vehicular environment with constantly changing vehicular topology, there is a constraint on the communication resource availability and also on the time for communication and solution determination. To address the challenges of the vehicular environment, we propose the technique of virtual negotiation by means of the so-called “virtual deals” which require actual communication only at the start and end of the route-allocation process. In a virtual deal, an agent does not actually communicate with other agents but only enacts the process of inter-agent communication in its “mind”. The negotiation entails proposals and counter-proposals and in CARAVAN, it is in the form of virtual deals which involves simple inter-agent exchange of resources (routes) to arrive at a better allocation with the final agreed solution being a sub-optimal solution. The types of deals considered in the algorithm are: ADD, SWAP and DROP deals, explained later.

Three categories of welfare strategies considered in CARAVAN are: (i) Social Welfare–it aims to increase the overall utility of the allocation; (ii) Rational Welfare–it aims to increase the individual utility of the agent in the allocation; and (iii) Mixed Welfare–it aims to increase individual as well as the overall utility of all the agents in an allocation.

It is also to be noted that although the formulation in (3) applies to the optimization problems, we adopt a satisficing approach in CARAVAN, which means that the resulting value of the equation, may or may not be optimal.

The final satisficing solution obtained by the agents is exchanged and the best allocation in terms of the utility value of the: (i) *allocation* is accepted for Social and Mixed Welfare strategies; and (ii) *poorest agent* for Rational Welfare strategy as explained in Section IV.

For selfish agents in a non-cooperative environment, Braess Paradox arises which indicates that adding an extra link between the source and destination may not necessarily benefit users [18]. This happens because the agents do not coordinate their route choices with other agents. However, CARAVAN involves cooperative and informed decision making; hence, adding an extra link would in fact benefit the network users as the vehicles will be spread more evenly along the network depending on the cost of a route allocation and the agent preferences. We also note that the problem of allocating routes to vehicles has been well-studied using user equilibrium concepts and system-wide perspectives, but we approach the issue from a multiagent perspective, where cooperation among agents is localized and opportunistic (only if and when they meet at junctions), and not global, and investigate the large scale (i.e., global) effects of such local peer-to-peer style cooperation.

## Design of multiagent based virtual negotiation mechanism in CARAVAN

This section describes the design rationale of the virtual negotiation mechanism used in CARAVAN and provides guiding principles we used in the design, which is based on the notion of acceptable deals.

The inter-agent negotiation in CARAVAN takes place in the form of virtual deals. The types of deals for the sample initial allocation *{v1-r1*, *v2-r2*, *v3-r3}*, where *ri* (*i*< = 3) denotes the route assigned to vehicle *vj* (*j*< = 3) are described below:

- *ADD Deal*: In an ADD deal, an agent virtually assigns itself one of its preferred routes. For example, *v1* assigns itself to route *r2*.
- *SWAP Deal*: In a SWAP deal, an agent exchanges its route with another agent. For example, *v1* and *v3* swap routes.
- *DROP Deal*: In a DROP deal, an agent assigns itself a route that has currently been assigned to some other agent, whereas the other agent is assigned another route from the available set of routes. For example, *v1* drops its route and is assigned the route of agent *v3*, and *v3* is assigned another random route *r1*.

A deal (ADD/SWAP/DROP) is said to be *acceptable* if the difference in the utilities of the final allocation (allocation resulting from the application of the deal) and the initial allocation is greater than or equal to zero. The difference in utilities is the sum of the difference in the aggregate preference and the difference in the aggregate cost for all the agents in the allocation. If a deal between agents *i* and *j* in an initial allocation *σ* results in a final allocation *σ’*, and *Δ* denotes the difference in values (preference utility index and cost), i.e. the difference in the final utility *u(σ')* and the initial utility *u(σ)* of the allocation, the overall difference in utility for the allocation can be represented as follows:

$$\Delta (u)=u(\sigma^{\prime })-u(\sigma )$$ (4)

The value of *Δ(u)* is necessarily positive because only those deals which result in increase in the utility value are accepted.

In [1], CARAVAN was evaluated for Social, Rational and Mixed Welfare types.

The aggregate difference in the utility *(Δ(u))* for Social and Mixed Welfare is the sum of: (i) the difference in the aggregate preference utility index value of the agents *i* and *j* involved in the deal, denoted as: Δ*p*_*i*_ + Δ*p*_*j*_; and (ii) the difference in the aggregate cost for all the agents, denoted as: $\sum_{k=1}^{n}\Delta c_{k}$. It is represented as follows:

$$\Delta (u)=\Delta p_{i}+\Delta p_{j}+\sum_{k=1}^{n}\Delta c_{k}$$ (5)

A deal is said to be acceptable if the aggregate difference in the utility is greater than or equal to zero. It is represented as:

Δ(u)≥0 (6)

The aggregate difference in the utility (Δ(*u*_*i*_)) of agent *i* for Rational and Mixed Welfare, initiating the deal is the sum of: (i) the difference in its preference utility index value, denoted as Δ*p*_*i*_; and (ii) the difference in cost of an allocation for agent *i*, denoted as Δ*c*_*i*_. It is represented as follows:

$$\Delta (u_{i})=\Delta p_{i}+\Delta c_{i}$$ (7)

A deal is said to be acceptable for an agent if its utility in the resulting allocation is same or greater than its utility in the initial allocation.

$$\Delta (u_{i})\geq 0$$ (8)

Note that the deals help the participating agents increase the utility of their choice of routes since a deal (ADD/SWAP/DROP) is acceptable and only done if the difference in the utilities of the final allocation (i.e., the allocation resulting from the application of the deal) and the initial allocation is zero or more.

## Experiments and analysis of CARAVAN

In our previous work [1], the algorithm was tested for small and large synthetic and real-road networks for the three welfare types. The results compared the percentage reduction in travel time against the Shortest Path Algorithm. It was observed that the percentage reduction in travel time increased from single to large real-road networks as also with increase in the number of vehicles. It was 21–43% when the traffic was below the network capacity and 13–17% when the traffic was above network capacity. The travel time savings increased with the availability of the amount of real-time information than with just the static traffic information. It was consistent, around 23%-36% for the 5 scenarios with 80 vehicles and random source-destination pairs. The results demonstrate that the Rational and Mixed Welfare strategies perform as well as the Social Welfare strategy. For this reason, for the experimental evaluations presented in this paper we will be concentrating only on the Social Welfare strategy of CARAVAN.

In this paper, we test the robustness and scalability of the approach for different topologies and sizes of the real road networks such as a large real-road network and a grid network. The performance of the algorithm in terms of travel time reduction was evaluated over a range of: (i) environmental parameters, to test the effect of constituent entities in the traffic environment by varying the number and placement of junctions and the road segment capacity; (ii) algorithmic parameters, to test the effect of variations in the algorithmic entities used to evaluate the route preferences and allocation utility; and (iii) agent-related parameters, to test the effect of varying percentage of equipped vehicles and compliant drivers. The results are presented in the form of graphs that compare the performance of CARAVAN against the Dijkstra’s algorithm [19], which is one of the de-facto shortest path algorithms [20]. The performance of the algorithm in terms of aggregate travel time or percentage reduction in travel time was evaluated and analyzed for variations in the algorithmic, environmental and agent-related parameters. The performance of CARAVAN in terms of driver preference satisfaction is measured for variation in algorithmic parameters where applicable. The roads in this network are single-lane roads and run from left to right and have speed limits. Every junction splits into two or three alternate routes. CARAVAN was simulated using JADE as the agent simulator and VanetMobiSim as the mobility simulator. We clarify that negotiation happens at the junctions among vehicles that come near to each other in a dynamic way as the simulation progresses with the movement of cars—our simulation is dynamic, considering actual vehicles travelling from an origin to a destination, cooperating along the way with cars they come near to at junctions.

### Large real road network

**Fig 1:**
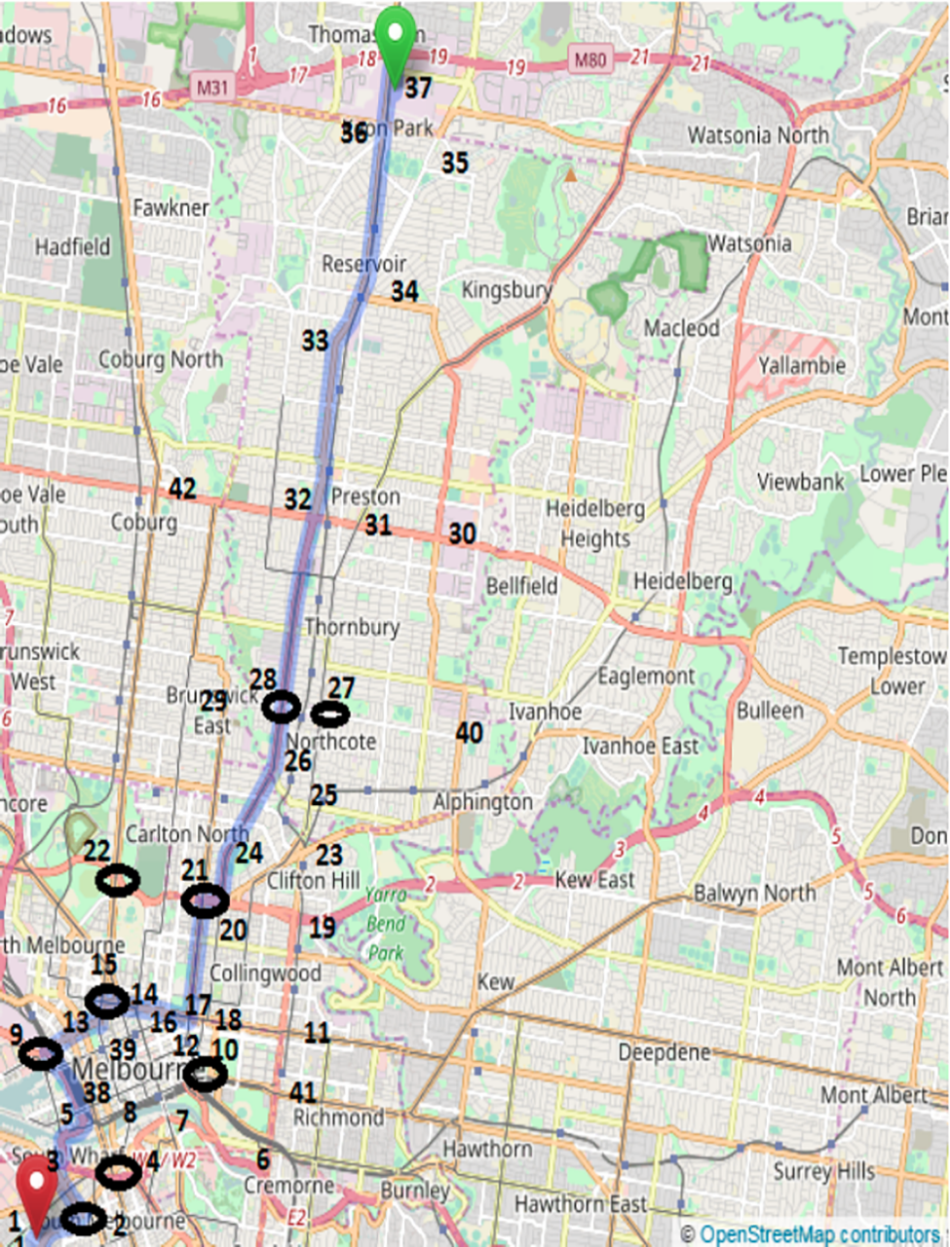
Large real-road network in Melbourne.

The large real-road network in Melbourne depicted in Fig 1 was used to further test the robustness of the algorithm for variations in different types of environmental, algorithmic and network parameters. For the purposes of simulation, the road length, and speed limits were reduced to scale while maintaining the original topology. The road network contains 10 junctions at nodes 2, 4, 9, 10, 15, 21, 22, 27, 28 and 34 (marked using ovals). At every junction, each vehicle agent applies twenty iterations of random deals (empirically found to be an effective number for generating efficient allocations). The experiments use combinations of 1 to 6 junctions as per the requirements of the scenario. The effect on the travel time and/or the preference utility index is shown in the form of a graph for each of the scenarios in the following sections.

### Variations in algorithmic parameters

**Fig 2:**
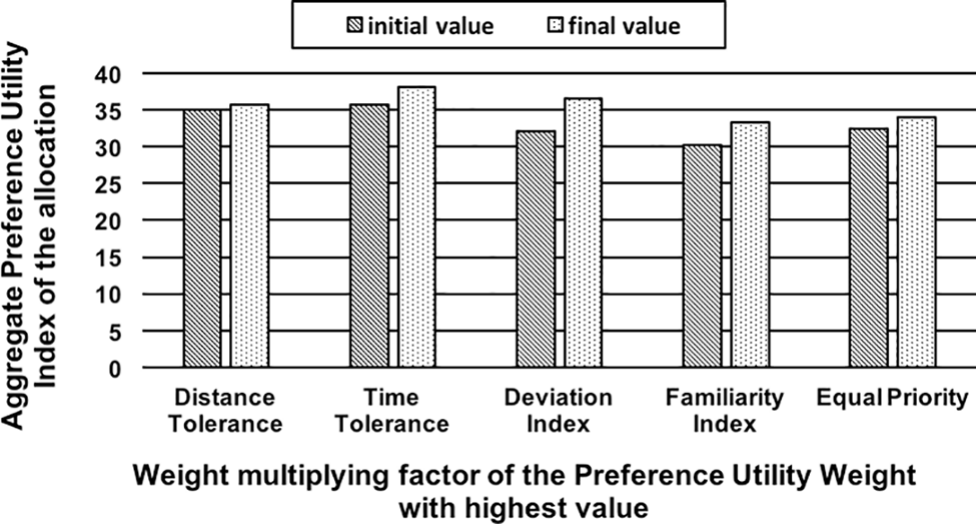
Effect of varying weight multiplying factors for the preference utility weight.

**Fig 3:**
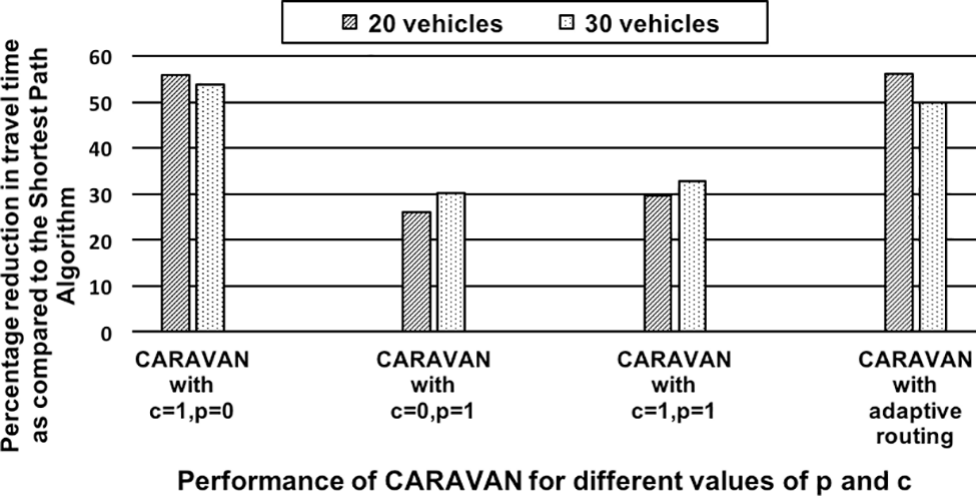
Percentage reduction in travel time over the Shortest Path Algorithm for a congested scenario.

We investigate the following parameters:

1. *Effect of Variations in the Preference Utility Weight Factors on Route Allocation*: In this scenario, the PUW multipliers were varied and their influence on the aggregate PUI of the initial and final allocations were compared. The scenario analyzes how the algorithm adapts to the varying significance attached to a preference criteria in the evaluation of Preference Utility Weight. The PUW of a vehicle for a particular route is calculated using the weighted combination of the following factors: Choice of Alternate routes, *a* (weight: 0.3), Previous compliance, *c* (weight: 0.3), Deviation Index, *i* (weight: 0.1), Time Tolerance, *t* (weight: 0.1), Distance Tolerance, *d* (weight: 0.1), Familiarity Index, *f* (weight: 0.1). The choice of the values assigned to the weights can vary, and we study and compare five cases of variations below, each highlighting the importance of a particular aspect. In this scenario, these weight multiplying factors are varied and the aggregate value of the PUI from the initial and final allocations was compared. For this scenario, 75% of the vehicles are configured with Shortest Time as their primary preference criteria and the remaining 25% with Shortest Distance as their primary preference criteria. The simulation is performed for a single junction 20-vehicle scenario and for non-zero values of both *p* and *c*. The results for the effect of variations in the PUW multiplying factors are summarized as below and the results are compared in Fig 2.
  - *Equal Priority Case* with *a*, *c*: 0.3 and *i*, *t*, *d*, *f*: 0.1—The algorithm tries to improve the utility of the allocation by allocating as many vehicles as possible to the primary preference (most desired) route type i.e. Shortest Time route, while also not compromising on the preferences of the other vehicles to a large extent. The number of vehicles switching to their preferred routes is seven.
  - *Familiarity Index* priority case with *c*, *f*: 0.3 and *a*, *i*, *t*, *d*: 0.1—The “familiar” routes carries more weightage (i.e. is the most preferred route type). The number of vehicles on their familiar routes in the final allocation improves from six in the initial allocation to ten in the final allocation.
  - *Deviation Index* case with *c*, *i*: 0.3 and *a*, *i*, *t*, *d*: 0.1—The algorithm tries to improve the utility of the allocation by allocating the vehicles to their primary or secondary route choices. Higher weightage to the deviation index (or *devindx*, for short) factor indicates that the vehicles are less flexible to deviate from their route choices. “Shortest Time” route type is the most preferred route choice. The results show that five vehicles switch to their preferred route type.
  - *Time Tolerance* case with *c*, *t*: 0.3 and *a*, *i*, *d*, *f*: 0.1—The “Shortest Time” routes carries more weightage (i.e. is the most preferred type of route), the algorithm tries to improve the utility of the allocation by allocating the vehicles to the Shortest Time route. The results show that the number of vehicles which have switched to their preferred route type of ‘Shortest Time’ has increased to nine from five.
  - *Distance Tolerance* case with *c*, *d*: 0.3 and *a*, *i*, *t*, *f*: 0.1—The “Shortest Distance” routes carries more weightage (i.e. is the most preferred type of route), there was a gain in PUI values. However, there was no new switch to the Shortest Distance route by any vehicle, as the initial allocation already had preferred routes assigned to those vehicles with Shortest Distance route preference.
    It is observed that for each of the settings, the final aggregate PUI value resulting from the application of the algorithm was better than the initial value. The results also show that the vehicles switch to their preferred route type according to the weightage carried by that route type.
2. *Effect of Variation in Cost and Preference Multipliers on Travel Time Reduction for Congested Segments*: The scenario demonstrates the ability of the algorithm to adapt its route allocation to maximize the travel time savings for different values of preference and cost multipliers in case of congested segments. This scenario is simulated for a set of 20 vehicles (source nodes 2) and 30 vehicles (source nodes 2 and 41) and 8 junctions (nodes 4, 9, 10, 15, 22, 27, 28 and 34) with three congested road segment. The vehicles travel at lowest speed on these congested segments. This scenario assumes that information about the congested road segments is available to the vehicles. The scenario was simulated for different values of *p* and *c*: (i) *c* = 1 and *p* = 0: the vehicles completely avoid the congested road segments by adaptively routing to another route; (ii) *c* = 1 and *p* = 1: the vehicles try to avoid the congested road segment as much as possible. However, as the value of *p* is 1, the vehicles also try to follow their preferred routes; (iii) *p* = 1 and *c* = 0: the vehicles only follow their preferred routes irrespective of the few of them being congested; and (iv) adaptive routing: the algorithm adaptively ignores the route preferences in cases where there is a possibility of the vehicle going on to a congested route without the further possibility of re-routing. For each of the cases, percentage reduction in travel time against the Shortest Path Algorithm was recorded. As shown in the graph in Fig 3, the results overall suggest that for all the combinations of values of *p* and *c*, CARAVAN offers considerable reduction in travel time as compared to the Shortest Path Algorithm.
  For the cases where CARAVAN is simulated with value of *c* set to 0 and *p* set to 1, there is the lowest percentage reduction in terms of travel time. This is because the agents entirely follow their preferences in spite of the forthcoming congested segments. In such cases, if CARAVAN switches the value of *c* to 1, the percentage reduction in travel time obtained increases by up to about 4%. Furthermore, if the value of *p* is set to 0 (preferences not taken into consideration), the savings obtained by reduction in travel time further increases by around 21–26%. For the case of adaptive routing, as CARAVAN selectively takes the route preferences into consideration and ignores them otherwise, the travel time savings is 50–56% more than the Shortest Path Algorithm. The flexibility in tuning the algorithmic values proactively avoids the congested segment and adapts the route allocation. As can be seen from the graph, this improves the network performance resulting in reduction in the travel time.

### Variations in environmental parameters

**Fig 4:**
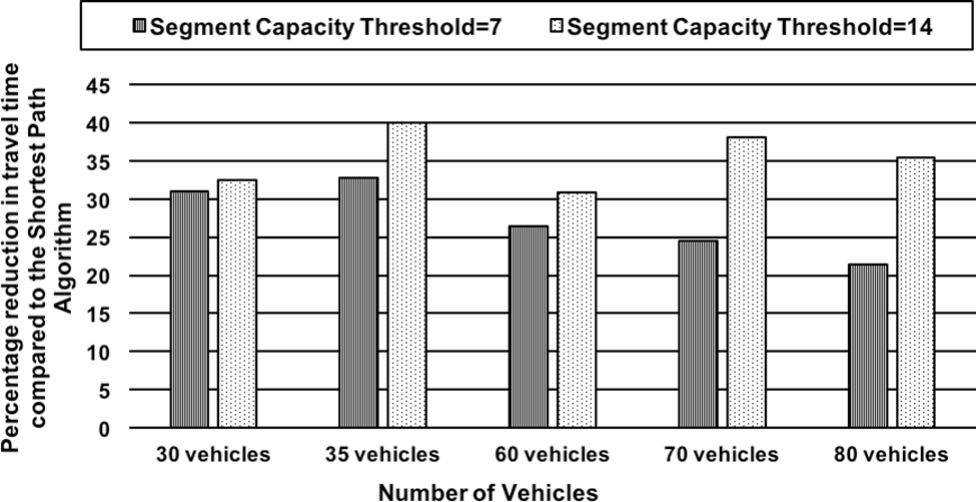
Effect of segment capacity threshold on percentage reduction in travel time over the Shortest Path Algorithm.

**Fig 5:**
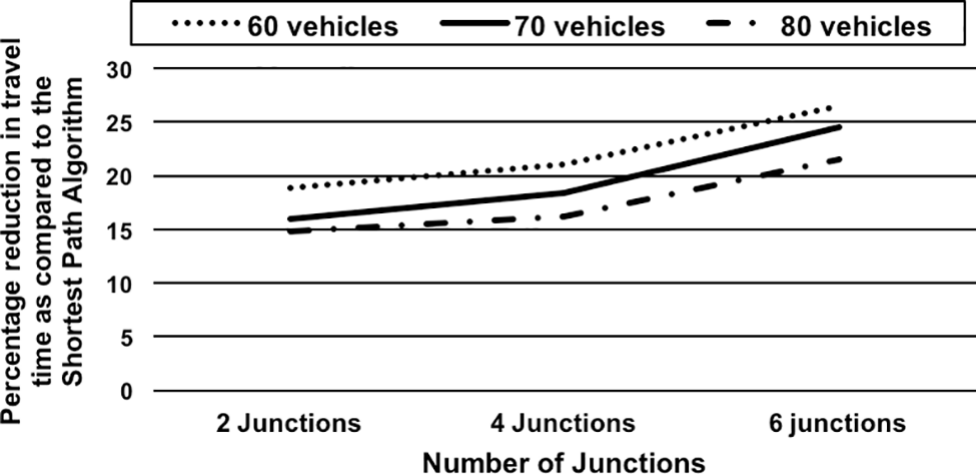
Effect of Number of junctions on percentage reduction in travel time over the Shortest Path Algorithm.

We investigate the following parameters:

1. *Effect of Road Segment Threshold Capacity on Travel Time*: The scenario demonstrates the effect of variations in segment threshold capacity on CARAVAN (practical / threshold capacity of a road is the number of vehicles that can traverse the road at free-flow speed per unit time, and beyond which congestion starts to build up). In this scenario, small and large sets of vehicles from 30 to 80 vehicles are considered for simulation. Six junctions (nodes 2, 4, 9, 10, 15 and 22) are used for the simulation. For the sets of 30 and 35 vehicles, all of them start from one source node. For the set of experiments with 60, 70 and 80 vehicles, the first 35 vehicles start from source A (node 1)–they encounter 6 junctions till they reach the destination and the rest of the 25, 35 and 45 vehicles, respectively start from the other source node (node 8)–they encounter only 4 junctions till they reach the destination. The average difference between the segment capacity and threshold is about 7 for the network. In this scenario, the segment capacity threshold capacities are incremented by counts of 7 and 14 and the effect on percentage reduction in travel time over the Shortest Path Algorithm using CARAVAN is observed and depicted using the graph in Fig 4.
  The graph shows that for threshold capacity increments of 7 and 14, the percentage reduction in travel time is on an increasing trend for the cases of 30- and 35-vehicle sets (as all vehicles encounter 6 junctions) and decreases further from 60- to 80-vehicle sets (as most of the vehicles encounter only 4 junctions). With the lesser opportunities to negotiate, the percentage reduction in travel time obtained drops after the 35-vehicle case. For the case with threshold capacity increment of 7, the percentage reduction in travel time dropped for the set of 60 vehicles as the network reaches its threshold capacity. It decreased further for the 70 and 80 vehicles sets. For the threshold capacity increment of 14, the percentage reduction in travel time obtained drops for the 60-vehicle set and again increases for the 70-vehicle set which is the network threshold capacity and hence thereafter decreases for the set of 80 vehicles. For the case with lower value of threshold increment (value of 7), congestion sets in earlier for higher vehicle sets (60 to 80) and hence percentage reduction in travel time compared to the Shortest Path Algorithm is less. However, when the segment capacity threshold increment value is 14, the saturation point of the network shifts further up as more vehicles can travel at free-flow speed.
2. *Effect of Varying the Number of Junctions on Travel Time*: This scenario demonstrates the significance of repetitive cooperative decision-making on the performance of CARAVAN in terms of percentage reduction in travel time by varying the number of junctions (decision-making points). In this scenario, the simulation is carried out for a 60, 70 and 80 vehicle set. For each of the vehicle sets, the number of junctions on the path is varied from 2 (nodes 2 and 4), 4 (nodes 2, 4, 15 and 27) and 6 (nodes 2, 4, 9, 10, 15 and 27). The percentage reduction in travel time as compared to the Shortest Path algorithm obtained for each case is depicted in the form of a graph in Fig 5. The results show that the percentage reduction in travel time as compared to the Shortest Path algorithm increases, with the increase in the number of junctions. The greater the number of decision points (junctions), the greater the opportunity vehicles get to negotiate and make cooperative routing decisions. This allows the vehicles to be distributed more evenly along the routes resulting in better travel times.

### Variations in agent-related parameters

**Fig 6:**
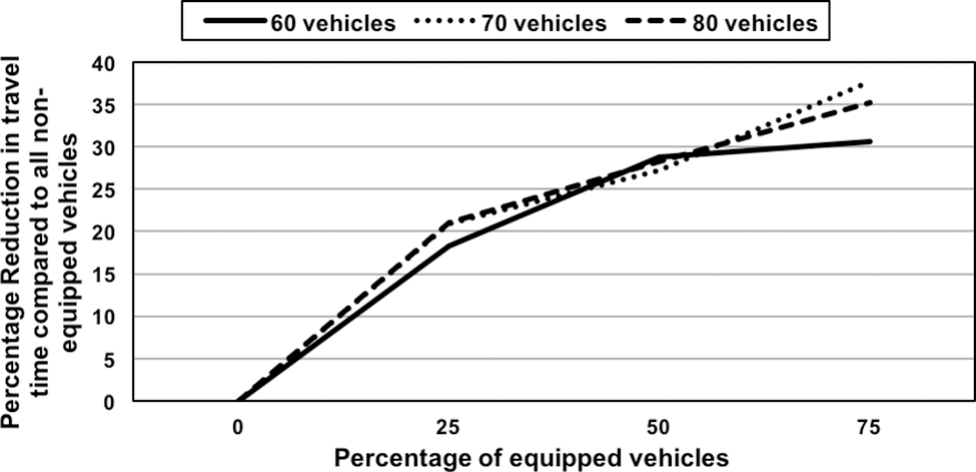
Effect of varying percentage of equipped vehicles on travel time reduction.

We investigate the effect of non-equipped vehicles on travel time. The scenario demonstrates the significance of cooperative route allocation in CARAVAN by varying the percentage of equipped vehicles. In this scenario, the simulation was carried out for 60, 70 and 80 vehicle sets. For each of the vehicle sets, the percentage of equipped vehicles is varied from 0−75%. When none of the vehicles are equipped, all the vehicles take the Shortest Path. The effective travel time for each of the cases with varying percentage of equipped vehicles is compared to the time obtained with the scenario where none of vehicles are equipped and the percentage difference in travel time is computed. The simulation results are shown in the form of a graph in Fig 6. For example, in the case of 25% equipped vehicles, the overall travel time reduction is 18–21% higher than the overall travel time obtained when none of the vehicles are equipped. For 50% or more of the equipped vehicles, the overall travel time reduction is 27–37% higher than the overall travel time when none of the vehicles are equipped. The results show that even with the penetration rate of 25% (75% non-equipped vehicles), CARAVAN offers travel time gains outperforming the Shortest Path Algorithm.

### Real-road grid network

**Fig 7:**
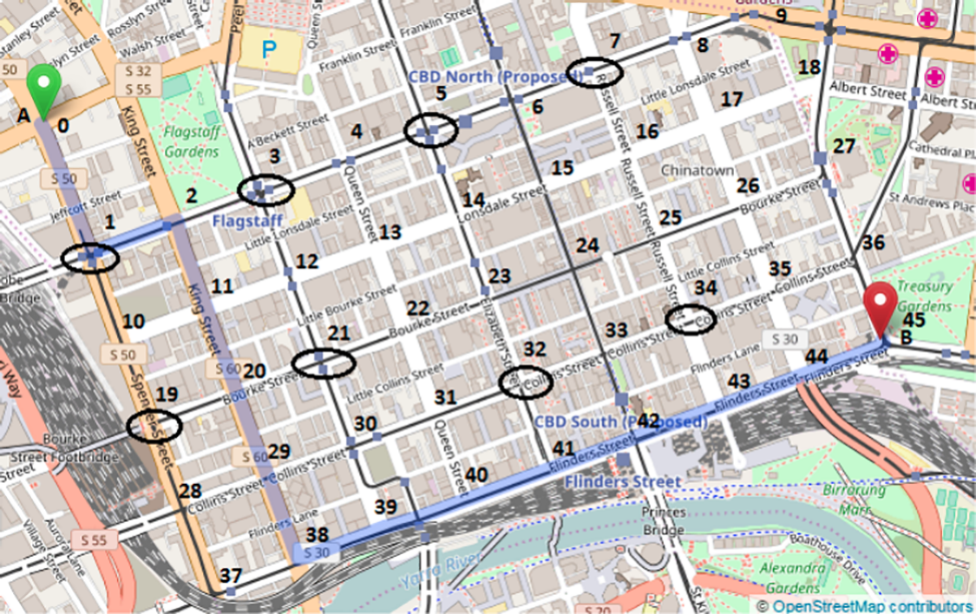
Real road grid network.

To validate the stability and scalability of CARAVAN, it was applied to an 8x4 real grid network with 80 vehicles as shown in Fig 7. The network comprises of the busier road segments in Melbourne Central Business District. The network contains 8 junctions as marked by an oval in the figure below (nodes 1, 3, 5, 7, 19, 21, 32 and 34). The scenarios were tested with various source nodes and with destination node 45 (‘B’). We expect similar results on the algorithmic parameters as that with the real-world network, and focus here on the effects of varying environmental and agent-related parameters, since the grid network offers a different topology and tends to increase the intensity of agent negotiations (i.e., more cooperation).

### Variations in environmental parameters

**Fig 8:**
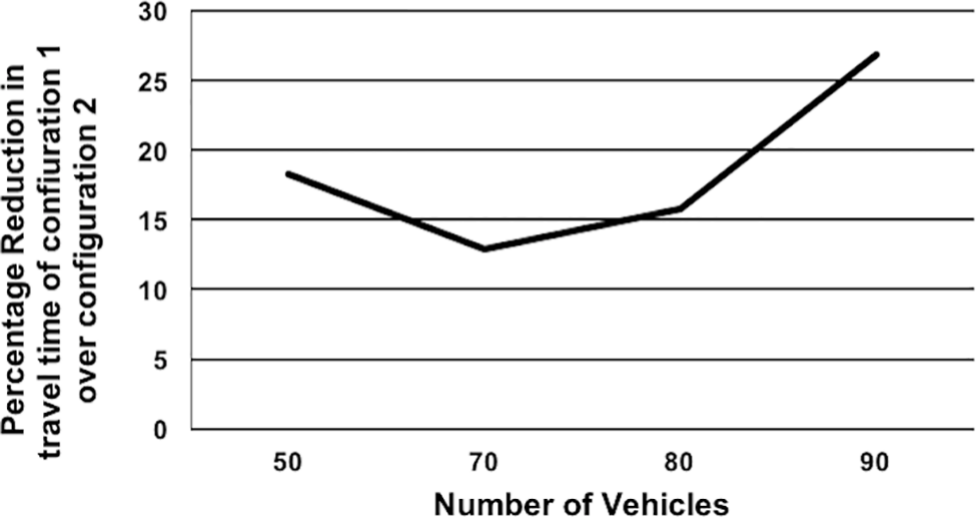
Effect of spatial distribution of junctions.

**Fig 9:**
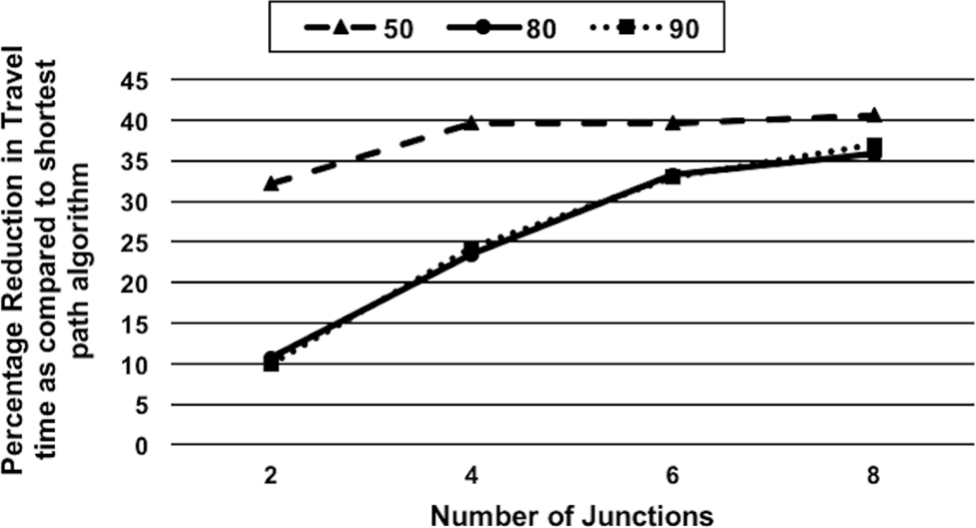
The effect of varying number of vehicles (50, 80 and 90) and number of junctions on travel time.

We investigate the following parameters:

1. *Effect of Varying Spatial Distribution of Junctions on Travel Time*: This scenario demonstrates the effect of junction placement on the travel time savings with CARAVAN. In this scenario, the percentage travel time was compared for 2 configurations of the road junctions. In configuration 1, all the junctions are in the beginning of the journey i.e. junctions 1,19,3 and 21 in Fig 7 and in configuration 2, there are 2 junctions each at the beginning and end of the journey i.e. junctions 1,19,7 and 34 in Fig 7. The simulation was carried out for sets of 50 to 90 vehicles. For the 70-vehicle set, nodes 0 and 2 are the source nodes. For the 80- and 90-vehicle set, nodes 0 and 3 are the source nodes. For the 50-vehicle set, 0 is the source node.
  Fig 8 depicts the gain in travel time obtained with configuration 1 over configuration 2. The results in Fig 8 indicate that the sooner the junctions are encountered in the journey, the better is the travel time. With configuration 1, as the vehicles encounter the junctions during the initial part of the journey rather than later, the vehicles can distribute themselves efficiently during the initial part of the journey itself. The vehicles in this case take less time to reach the destination than with configuration 2, where the vehicles encounter 50% of the junctions only during the later part of the journey. This indicates that the more the number of junctions (decision points) earlier on in the journey, the vehicles get a chance to negotiate at the right time and distribute efficiently along the routes. When the junctions are encountered in the later part of the journey, the vehicles get a chance to re-distribute themselves along the routes rather late. They might already have congested a few routes until then, thereby taking more time to reach the destination.
2. *Effect of Varying the Number of Junctions on Travel Time*: This scenario demonstrates the significance of frequent cooperative decision-making in CARAVAN for travel time savings by varying the number of junctions. In this scenario, the number of junctions is varied while keeping the number of the vehicles the same. This scenario is tested for each of the sets of 50, 80 and 90 vehicles with 2, 4, 6, and 8 number of junctions and with nodes 0 and 3 as source nodes. Each of the travel times obtained was compared with the Shortest Path Algorithm and represented using graph in Fig 9. Simulation results indicate that with the increase in the number of junctions, the total travel time decreases as the vehicle agents get more opportunities to negotiate, which distributes them evenly along the road networks. It is also observed that for each of the sets of 50, 80 and 90 vehicles, the variation in percentage reduction in travel time, between the 6-junction and 8-junction scenario is 1 to 4%. This is because the percentage reduction in travel time obtained with the increase in the number of junctions almost starts stabilizing at the junction count of 6.

### Variations in agent-related parameters

**Fig 10:**
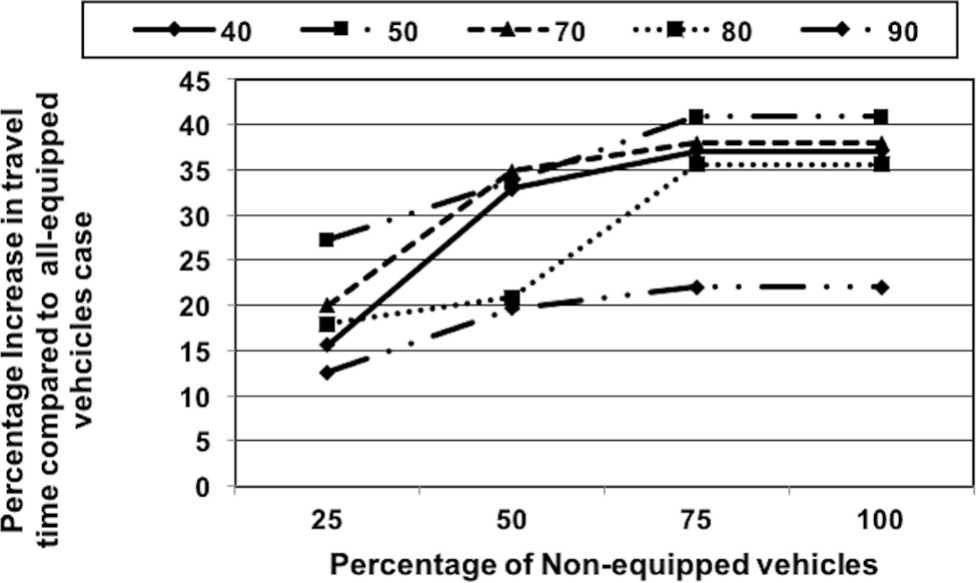
Effect of non-equipped vehicles—comparison of travel time with all-equipped vehicles.

**Fig 11:**
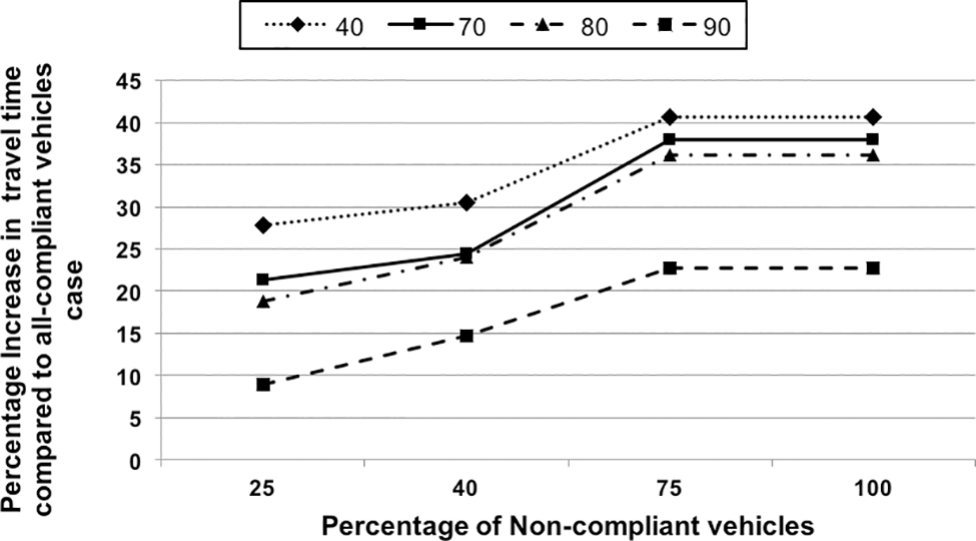
Effect of non-compliant vehicles—comparison of travel time with all-compliant vehicles.

We investigate the following parameters:

1. *Effect of Non-Equipped Vehicles on Travel Time*: This scenario demonstrates the significance of cooperative route allocation in CARAVAN by varying the percentage of equipped vehicles. In this scenario, the percentage of non-equipped vehicles was varied from 25–100% for sets of 40 to 90 vehicles with nodes 0 and 3 as the source nodes. 100% non-equipped vehicles indicate that none of the vehicles are equipped and they take the shortest path. The effective travel time for each of the scenarios was compared with the time obtained with the scenario where there are 100% equipped vehicles and the percentage difference in travel time was computed. The results are depicted in Fig 10. The simulation results show that as the percentage of non-equipped vehicles increases from 25% to 100%, the difference in travel times between the all-equipped and given percentage of non-equipped vehicles increases. This indicates that the savings in travel time reduce with the increase in the number of non-equipped vehicles. For example, in the case of 50% of non-equipped vehicles, the overall travel time is higher by about 20–34% than the overall travel time with the all-equipped case. This trend continues until the case with 75% non-equipped vehicles. For 75% or more non-equipped vehicles, the overall travel time is over 22–40% higher than that with the all-equipped case. It remains constant from 75–100% of non-equipped vehicles indicating that the effect with the 75% non-equipped vehicles is same as the one when none of the vehicles are equipped. Even with 50% non-equipped vehicles, the reduction in travel time obtained is better than the Shortest Path Algorithm (100% non-equipped vehicles). Overall, the data series trend lines show that the percentage reduction in travel time obtained increases from 40-vehicle to 50-vehicle set and then reduces as the vehicle count approaches the average network capacity from 70-vehicle to 90-vehicle set.
2. *Effect of Non-Compliant Vehicles on Travel Time*: The scenario demonstrates the significance of cooperative route allocation in CARAVAN by varying the percentage of compliant vehicles. In this scenario, the percentage of non-compliant vehicles is varied from 25–100% for sets of 40 to 90 vehicles with nodes 0 and 3 as source nodes. 100% non-compliance indicates that all the vehicles are non-compliant and they take the shortest path. The effective travel time for each of the scenarios was compared with the time obtained with the scenario where there are 100% compliant vehicles and the percentage difference in travel time was computed.
  The simulation results in Fig 11 show that as the percentage of non-compliant vehicles is increased from 25, 40, and 75 to 100%, the difference in travel times of the all-compliant and (given percentage of) non-compliant vehicles increases. This indicates that the savings in travel time reduce with the increase in the number of non-compliant vehicles. For example, in the case of 40% non-compliant vehicles, the overall travel time is higher by about 15–30% than that with the all-compliant case. This trend continues until with 75% of non-compliant vehicles, the overall travel time is over 22–40% higher than the overall travel time with the all-compliant case. It remains constant from 75–100% of non-compliant vehicles indicating that the effect with the 75% non-compliant vehicles is same as the one when none of the vehicles are compliant. The results show that even with 50% non-compliant vehicles, the reduction in travel time obtained is better than the Shortest Path Algorithm (100% non-compliant vehicles). Overall, as the vehicle count approaches the average network capacity, the data series trend lines show that the percentage reduction in travel time obtained reduces from 40-vehicle set to 70-vehicle and then further up to 90-vehicle set as the vehicle count approaches the network capacity.

We note that a vehicle being non-compliant is different from one being non-equipped, since the preferences of non-compliant vehicles are involved in the CARAVAN route allocation algorithm (even if the non-compliant vehicles are not following CARAVAN allocated route choices).

### Summary of results

Table 1 summarizes the experimental results.

**Table 1:** Summary of experimental results and conclusion.

Scenario Description	Summary of Results	Conclusion
***Algorithmic Parameters***		
Variation in PUW Factors	Allocation obtained varies in favor of the preference type with higher weight.	The algorithm adapts to the changes in the type of route.
Preference and Cost Multiplier variations	The scenario with *c* = 1, *p* = 0 gives highest reduction in travel time. Even for congested road segments, CARAVAN offers considerable reduction in travel time compared to the Shortest Path Algorithm.	This shows the ability of CARAVAN to do proactive, reactive and adaptive routing.
***Environmental Parameters***		
Number of Junctions	There is a greater percentage reduction in travel time with an increase in the number of junctions.	With greater number of junctions, vehicles get more opportunities to negotiate, leading to more evenly distributed traffic, reducing the aggregate trip time.
Spatial Distribution of Junctions	Percentage reduction in travel time for the first four junctions is better than that for the distributed junctions.	The sooner the vehicle get an opportunity to negotiate, the better is their distribution along the road network
Segment Threshold Capacity	The percentage reduction in travel time obtained improves as the threshold capacity value increases. The saturation point of the network shifts with the increase in the Segment Capacity Threshold.	CARAVAN can give more effective gains for wider roads with higher capacities.
***Agent-related Parameters***		
Non-equipped vehicles	Percentage reduction in travel time obtained with CARAVAN reduces as the number of non-equipped vehicles increases. For large real road network, CARAVAN offers travel time savings with just 25% penetration rate.	In terms of reduction in travel time, CARAVAN outperforms the Shortest Path Algorithm even for 50% equipped vehicles.
Non-compliant vehicles	Percentage reduction in travel time obtained with CARAVAN reduces as the number of non-compliant vehicles increases.	In terms of reduction in travel time, CARAVAN outperforms the Shortest Path Algorithm even for 50% compliant vehicles.

## Conclusion

This paper has presented a comprehensive evaluation of CARAVAN for different types of road networks and analyzed its performance in terms of travel time reduction for a variety of parameter settings. We observed that effective global behaviors can emerge from local negotiation regulated by an appropriate combination of individual behavior, local group interaction, and environmental factors. We also observe that a cooperative vehicles approach can be robust across different variations in participation, road network structure and agent behaviours.

Much future work remains. For example, we investigated the scenario of having some non-equipped vehicles among equipped vehicles–a likely scenario in the near future, where there will be some vehicles not equipped and some equipped, during a transition phase where uptake could be gradual; we showed the effect on the travel time of all the vehicles (equipped or not) in the experimental results. One intuition is that having non-equipped vehicles among equipped vehicles will reduce the extent of negotiation (or cooperation) for all vehicles (whether equipped or not) and they will be affected just as much. But the question remains as to whether equipped vehicles will still be better off than non-equipped vehicles, or what proportion of vehicles at junctions will need to cooperate (and how often) in order to get travel time savings. Also, the results might vary depending on the distribution of equipped and non-equipped vehicles in the mix and their intended routes—much more experimentation is required to investigate these “transition” scenarios.
